# Metabolic Syndrome in Post-Pulmonary Tuberculosis-Associated Obstructive Airway Disease: A Cross-Sectional Analytical Study

**DOI:** 10.7759/cureus.23640

**Published:** 2022-03-30

**Authors:** Shahana MP, Madhusmita Mohanty Mohapatra, Vemuri Mahesh Babu, Manju Rajaram, Sathish Rajaa, Senthil Kumar Gandhipuram Periyasamy

**Affiliations:** 1 Pulmonary Medicine, Jawaharlal Institute of Postgraduate Medical Education & Research, Puducherry, IND; 2 Community Medicine, Employee's State Insurance Corporation Medical College & Post Graduate Institute of Medical Science and Research, Chennai, IND; 3 Biochemistry, Jawaharlal Institute of Postgraduate Medical Education & Research, Puducherry, IND

**Keywords:** tuberculosis-associated obstructive airway disease, endocrinology and diabetes, waist circumference, tuberculosis, metabolic syndrome

## Abstract

Background

The aim of this study is to assess the prevalence of metabolic syndrome in tuberculosis-associated obstructive airway disease (TOPD) patients, as well as the association of its components with the severity of airflow obstruction.

Methodology

In this cross-sectional analytical study, we evaluated the clinical profile, spirometry, waist circumference, blood pressure, lipid profile, fasting plasma glucose, and the association of each component with the severity of airflow obstruction.

Results

The prevalence of metabolic syndrome in TOPD was found to be was 25.77% (95% confidence interval = 18.11-35.28) among study participants. Reduced high-density lipoprotein was the deranged component and was associated with increased severity in Global Initiative for Chronic Obstructive Lung Disease (GOLD) stage II compared to GOLD stage IV.

Conclusions

The prevalence of metabolic syndrome in TOPD has a severe impact on patients’ treatment, outcomes, and complications. However, in our study, tuberculosis-associated metabolic syndrome was the same as the general population. Low high-density lipoprotein levels were associated with the severity of the airflow obstruction.

## Introduction

Tuberculosis (TB), a disease of old times, has remained an important infectious disease globally. Around 10 million new cases of TB occur globally every year, and 1.2 million deaths occur due to TB in human immunodeficiency virus (HIV)-negative people. India (26%) is among the eight countries that account for two-thirds of the global TB burden [1]. According to estimates, roughly, of every five Indians, two people are infected with TB, and about 3,22,000 people die every year of TB in India [2]. Chronic obstructive pulmonary diseases (COPD) cause a substantial impact on morbidity and mortality worldwide. It is estimated that the prevalence of COPD was 251 million individuals in 2016, with 3.17 million deaths in 2015; 90% of deaths occur in low- and middle-income countries [3]. An increasing number of smokers, pollution in developing nations, and the rise in the elderly population in low-income nations have contributed substantially to the increase in the incidence of COPD patients. It is estimated that COPD will be the underlying cause of 7.8% of deaths by 2030 [4]. TB and COPD are the two important causes of morbidity and mortality in India. They remain among the top 10 causes of death in India [5].

Metabolic syndrome, otherwise known as syndrome X, is a novel clinical challenge, and various clinical criteria have been framed to define metabolic syndrome [6]. The National Cholesterol Education Program (NCEP) Adult Treatment Panel III (ATP III) devised a definition for metabolic syndrome, as characterized by the findings of central obesity, high blood pressure (BP), raised triglyceride (TG) levels, low high-density lipoprotein cholesterol (HDL-C) levels, and raised fasting plasma glucose (FPG) levels. The American Association of Clinical Endocrinology modified ATP III by considering insulin resistance as the core element. The International Diabetes Federation (IDF) defines central obesity using any two criteria, namely, raised BP, raised FPG, raised TG, and low HDL-C [7]. Metabolic syndrome is common in patients with COPD. Compared to the general population, metabolic syndrome is twice more common in COPD patients, and its prevalence among COPD patients varies from 25.6% to 60.9% in the literature [8]. Tuberculosis-associated obstructive airway disease (TOPD) forming the TOPD phenotype of COPD is a sequela and complication following tuberculosis, and a considerable number of COPD patients belong to this group [9]. Because metabolic syndrome is common in COPD patients, we expected that there might be a higher prevalence of metabolic syndrome and its components among TOPD patients. Hence, this study was conducted under this assumption.

## Materials and methods

The aim of the study is to detect the prevalence of metabolic syndrome in TOPD patients, as well as the association of its components with the severity of airflow obstruction.

A cross-sectional analytical study was conducted in the Department of Pulmonary Medicine of a tertiary care center in South India from January 2018 to January 2020. Patients with a history of pulmonary TB for not less than one year were selected. Patients who were clinically stable, diagnosed with post-pulmonary tuberculosis with spirometry, diagnosed with COPD as per the 2017 Global Initiative for Chronic Obstructive Lung Disease (GOLD) criteria, and aged ≥40 years were included in the study [10]. People with interstitial lung disease, lung cancer, psychiatric illness, alcoholics, pregnant women, and those with pre-existing co-morbidities, such as gastroesophageal reflux, hypertension, and diabetes mellitus, were excluded from the study. Both current smokers and patients with a biomass fuel exposure index of more than 60 were also excluded from the study as they were potential confounders.

The sample size was calculated using the estimation of a population proportion. With a 95% confidence level (CI) and taking the prevalence of metabolic syndrome in COPD patients as 46% using the modified NCEP ATP III criteria, as per the study by Acharyya et al. [11]. Hence, presuming that metabolic syndrome in TOPD patients was the same as COPD patients at around 50% with an absolute precision of 10% and attrition of 10%, the final sample size was calculated at 97.

Patients with a diagnosis of TOPD who were attending the Pulmonary Medicine Outpatient Department during the study period and met the inclusion criteria were enrolled in the study. Prior Institutional Ethical Clearance was obtained and written informed written was taken from patients during enrolment. Patients were initially evaluated for COPD using the COPD Assessment Test (CAT) questionnaire. Information regarding age, gender, education level, occupation, smoking status, and alcohol intake was recorded during a face-to-face interview in a prerequisite proforma. Patients’ smoking status was registered as never smokers, former smokers, and current smokers, along with details of pack years for those who had smoked. Never smokers were people who had never smoked or had smoked less than 100 cigarettes in their lifetime. Former smokers were adults who had smoked at least 100 cigarettes in their lifetime and had quit smoking at the time of the interview. Current smokers were adults who were still smoking at the time of the interview. The biomass fuel exposure index of patients was also recorded. The biomass fuel exposure index was calculated as the average number of hours spent on cooking daily multiplied by the total number of years spent on cooking personally [12]. An index of 60 was considered significant. Hence, patients with a biomass fuel exposure index of less than 60 and a history of pulmonary TB were also included.

Once recruited, a chest X-ray (CXR) posterior-anterior view was taken to detect any evidence of post-pulmonary TB sequelae and hyperinflation. Two sputum samples for acid-fast bacilli by smear were sent for investigation to exclude microbiologically active cases of pulmonary TB. Spirometry was done and post-bronchodilator values with salbutamol solution 2.5 mL (each mL containing salbutamol equivalent to 5 mg) using an ultrasonic nebulizer were noted down. Based on post-bronchodilator forced expiratory volume in 1 second (FEV1), the study participants were categorized into four severity grades based on the GOLD 2017 criteria. A fasting blood sample of 5 mL was taken using an aseptic method with sodium fluoride as a preservative from each patient for fasting blood sugar and lipid profile. A mercury sphygmomanometer was used to measure blood pressure in the arm in a sitting position twice within five minutes. The waist circumference (WC) of study participants was measured using a measuring tape for assessing central obesity.

Statistical analysis was done using SPSS version 19.0 (IBM Corp., Armonk, NY, USA). Continuous variables such as age, smoking status, symptomatology, CXR findings, and disease severity were expressed as mean and standard deviation (SD). Categorical variables were defined as percentages and 95% CIs. Outcome measures for the primary objective, that is, the prevalence of metabolic syndrome, were expressed as percentages with 95% CI. Multivariate analysis was done to determine the association of various components of metabolic syndrome with the GOLD stage of COPD. A p-value of <0.05 was considered statistically significant.

## Results

A total of 400 patients with symptoms suggestive of COPD were screened, and 97 cases of TOPD who satisfied the inclusion criteria were enrolled. In our study, more than half of the patients (54.64%) belonged to the age group of 40-60 years with a mean age of 51.3 years (SD: ±12.63) (Table 1). The majority (56.70%) of the patients were males, and about one-third (32.99%) of males belonged to the never smoker category. The majority of the patients (52.58%) who developed TOPD provided a history of onset of COPD symptoms in less than 10 years. The majority of our TOPD patients (62.88%) belonged to GOLD stage III with FEV1 of 30-49%). Moreover, the most common CXR findings in our study participants (37.11%) was degree 3 based on the Willcox classification. No difference was seen based on age, gender, smoking status, biomass fuel exposure, and history of pulmonary TB in TOPD patients with metabolic syndrome and without metabolic syndrome. The CAT score, CXR findings, and the proportion of different GOLD stages of TOPD between patients with and without metabolic syndrome were also similar (Table 1).

**Table 1:** Comparison of sociodemographic and clinical characteristics of TOPD patients with and without metabolic syndrome (n = 97). TOPD: post-pulmonary tuberculosis-associated obstructive airway disease; CAT: COPD assessment test; PTB: pulmonary tuberculosis; GOLD: Global Initiative for Chronic Obstructive Lung Disease

Characteristics	Frequency N (%)	Presence of metabolic syndrome among TOPD	Absence of metabolic syndrome among TOPD	P-value
Gender				
Female	42 (43.30)	13 (30.95%)	29 (69.05%)	0.308
Male	55 (56.70)	12 (21.82%)	43 (78.18%)	
Age in years				
<40	18 (18.56)	4 (22.22%)	14 (77.78%)	0.094
41–60	53 (54.64)	18 (33.96%)	35 (66.04%)	
>60	26 (26.80)	3 (11.54%)	23 (88.46%)	
Smoking				
Present	32 (32.99)	10 (31.25%)	22 (68.75%)	0.387
Absent	65 (67.01)	15 (23.08%)	50 (76.92%)	
Biomass fuel exposure				
Present	26 (26.80)	8 (30.77%)	18 (69.23%)	0.496
Absent	71 (73.20)	17 (23.94%)	54 (76.06%)	
CAT score				
10–20	7 (7.22)	1 (14.29%)	6 (85.71%)	0.661
21–30	37 (38.14)	11 (29.73%)	26 (70.27%)	
31–40	53 (54.64)	13 (24.53%)	40 (75.47%)	
History of PTB				
<10 years	51 (52.58)	12 (23.53%)	39 (76.47%)	0.59
≥10 years	46 (47.42)	13 (28.26%)	33 (71.74%)	
Extent of radiological lesion				
Degree I	11 (11.34)	3 (27.27%)	8 (72.73%)	0.942
Degree II	28 (28.87)	8 (28.57%)	20 (71.43%)	
Degree III	36 (37.11)	8 (22.22%)	28 (77.78%)	
Others	22 (22.68)	6 (27.27%)	16 (72.73%)	
GOLD staging				
STAGE I	2 (2.06)	1 (50%)	1 (50%)	0.69
STAGE II	21 (21.64)	15 (71.43%)	6 (28.57%)	
STAGE III	61 (62.88)	45 (73.77%)	16 (26.23%)	
STAGE IV	13 (13.40)	11 (84.62%)	2 (15.38%)	

The prevalence of metabolic syndrome in TOPD patients was 25.77% (95% CI = 18.11-35.28) (Table 2). The most deranged component of metabolic syndrome was central obesity and low HDL-C level. WC in females was higher compared to males (p = 0.011). HDL-C, an essential component of metabolic syndrome, was lower in females compared to males, and this difference was statistically significant (p = 0.001) (Table 3).

**Table 2:** Prevalence of metabolic syndrome in TOPD patients (n = 97). TOPD: post-pulmonary tuberculosis-associated obstructive airway disease

Metabolic syndrome in TOPD patients	Number (N)	Percentage
Present	25	25.77%
Absent	72	74.22%

**Table 3:** Prevalence of different components of metabolic syndrome in TOPD patients (n = 97). TOPD: post-pulmonary tuberculosis-associated obstructive airway disease; HDL: high-density lipoprotein; SBP: systolic blood pressure; DBP: diastolic blood pressure

Components of metabolic syndrome	Male n (%)	Female n (%)	P-value
Waist circumference			
>80 cm in females and >90 cm in males	16 (29.09)	23 (54.76)	0.011
Triglycerides			
≥150 mg/dL	5 (9.09)	7 (16.67)	0.262
HDL-Cholesterol			
<40 mg/dL in men and <50 mg/dL in women	19 (34.55)	32 (76.19)	0.000
Blood pressure			
SBP ≥130 mmHg or DBP ≥85 mmHg or use of antihypertensive drugs	24 (43.64)	15 (35.71)	0.430
Fasting plasma glucose			
≥100 mg/dL or use of oral hypoglycemic agent	14 (25.45)	7 (16.67)	0.298

Patients with GOLD stages II/III/IV had a higher prevalence of metabolic syndrome and its components, such as elevated TGs and blood sugars, compared to GOLD stage I. However, the above associations were not statistically significant except for GOLD stage II, which was associated with 3.3 times the risk of reduced HDL-C compared to GOLD stage IV (p = 0.02) (Table 4).

**Table 4:** Association of the individual risk components of metabolic syndrome with GOLD staging among TOPD patients (n = 97). CI: confidence interval: GOLD: Global Initiative for Chronic Obstructive Lung Disease; HDL: high-density lipoprotein; PR: prevalence ratio

	PR	95% CI	P-value
Metabolic syndrome			
GOLD I	3.25	0.49-21.36	0.22
GOLD 2	1.85	0.43-7.86	0.40
GOLD 3	1.70	0.44-6.52	0.43
GOLD 4	1	Ref.	
Elevated waist circumference			
GOLD I	1.46	0.79-6.97	0.29
GOLD 2	2.47	0.85-7.14	0.09
GOLD 3	1.56	0.54-4.45	0.40
GOLD 4	1	Ref.	
Elevated triglycerides			
GOLD I	1	Ref.	
GOLD 2	1.23	0.12-12.33	0.85
GOLD 3	1.91	0.26-13.85	0.51
GOLD 4	1.78	0.21-12.78	0.67
Reduced HDL-cholesterol			
GOLD I	2.16	0.39-11.91	0.37
GOLD 2	3.30	1.18-9.16	0.02
GOLD 3	2.20	0.79-6.12	0.13
GOLD 4	1	Ref.	
Elevated fasting plasma glucose			
GOLD I	1	Ref.	
GOLD 2	0.82	0.21-3.11	0.77
GOLD 3	0.99	0.33-2.96	0.99
GOLD 4	0.78	0.11-2.14	0.68
Elevated blood pressure			
GOLD I	1.31	0.29-5.81	0.72
GOLD 2	1	Ref.	0.97
GOLD 3	0 .98	0.52-1.86	0.36
GOLD 4	1.41	0.67-2.96	

## Discussion

In this cross-sectional study, 97 TOPD patients were screened for metabolic syndrome. The prevalence of metabolic syndrome based on the modified NCEP ATP III criteria was 25.77% (CI = 18.11-35.28). The overall prevalence of TOPD according to the PLATINO study was 30.7% compared to controls, and the development of obstructive airway disease was an independent risk factor in patients following TB [13]. According to studies conducted in India, the prevalence of TOPD varies between 15% and 46% [9,14-16]. Aggarwal et al. reported that almost one-third of COPD patients (32.4%) had an associated history of TB. They also reported that young patients with few pack-years of smoking had airflow obstruction in TOPD patients similar to COPD [17].

 A systematic review on the prevalence of metabolic syndrome in COPD patients showed a higher prevalence of metabolic syndrome in 32% of COPD patients than in 30% of controls (p = 0.001) [18]. The prevalence of metabolic syndrome in COPD patients has varied among studies. This can be attributed to the criteria used to define metabolic syndrome, as well as the country and ethnicity of the population where the study was conducted [19-20]. A cross-sectional study by Acharyya et al. found that metabolic syndrome had a prevalence of 44%, 46%, and 31% according to NCEP ATP III, modified NCEP ATP III, and IDF criteria, respectively, among COPD patients [11]. A community-based survey by Harikrishnan et al. in urban and rural areas of South India showed that the prevalence of metabolic syndrome was 24% when NCEP ATP III criteria were used [21]. A cross-sectional study by Venugopal et al. using the IDF criteria found that metabolic syndrome is present in 39.5% (95% CI = 35.3-44.1) of the general population [22]. In our study, the prevalence of metabolic syndrome in TOPD patients is almost similar to metabolic syndrome in the general population in South India, and there was no difference compared to the study done in Puducherry by Venugopal et al.

The association of individual risk components of metabolic syndrome with GOLD staging among TOPD patients was also studied. TOPD patients with GOLD stages I/II/III had more prevalence of metabolic syndrome and its components, such as elevated WC and reduced HDL-C, when compared to patients with GOLD stage IV. Overall, in our study metabolic syndrome was not associated with the severity of airflow obstruction. However, the prevalence of metabolic syndrome was higher in the early stages of COPD from grade I to III. Specific individual components, however, were associated with the severity of airflow obstruction. Among the individual risk components of metabolic syndrome, low HDL-C was associated with GOLD stage II, and this association was statistically significant compared to GOLD stage IV. Mannino et al. showed that COPD patients with GOLD stage III-IV had a higher chance of developing diabetes, a component of metabolic syndrome with an odds ratio of 1.5 with a 95% CI of 1.1-1.9 [23]. The Nurses’ Health Study by Rana et al. was a prospective cohort study conducted over eight years that showed that the age-adjusted risk of type 2 diabetes mellitus in COPD patients had a 1.8 times higher risk than in those without it [24]. COPD is considered a chronic inflammatory disorder, with an increase in circulatory inflammatory markers irrespective of the severity of lung function impairment. Oh et al. found that TOPD patients have higher levels of systemic inflammatory markers than smokers’ COPD. Higher levels of systemic inflammatory biomarkers in TOPD patients imply that these patients are at an increased risk of systemic morbidities and metabolic syndrome [25]. Arora et al. observed that all lipid profile components were within the normal limits in COPD patients, and there was no statistical difference in lipid profile between mild, moderate, and severe COPD [26]. In contrast, Zafirova-Ivanovska et al. reported that in COPD patients hypercholesterolemia significantly increased as the disease severity progresses [27].

Our primary strength was that this study evaluated the burden of metabolic syndrome among TOPD patients in an Indian setting. One novel attempt to check the association of components of metabolic syndrome among TOPD patients to see their individual diagnostic importance of metabolic syndrome. Despite this, our study had certain limitations. First, this was a cross-sectional study, and, hence, causal relationships of associations cannot be commented. Second, because the systemic inflammatory response that is responsible for the development of MetS in TOPD is high in TOPD compared to COPD, we did not measure any such marker of inflammation. Third, we did not extract data on dosages of ICS used by the participants, which may confound the prevalence of metabolic syndrome in TOPD patients. Fourth, due to the coronavirus disease 2019 pandemic and the nationwide lockdown during the study period, further screening and recruitment of patients could not be done.

## Conclusions

Although TOPD patients have high systemic inflammatory markers and metabolic syndrome is expected to be higher in these groups of patients, our study shows that metabolic syndrome in TOPD patients is almost the same as the general population. However, certain components such as low HDL-C are associated with the severity of GOLD staging of the TOPD phenotype of COPD. Prospective longitudinal studies are required to understand the relationship between metabolic syndrome and TOPD.
